# A qualitative interview study of the attitudes toward reproductive options of people with genetic visual loss

**DOI:** 10.1002/jgc4.1601

**Published:** 2022-07-04

**Authors:** Sophie Redgrave, Alisdair McNeill

**Affiliations:** ^1^ Department of Neuroscience The University of Sheffield, Sheffield Clinical Genetics, Service Sheffield Children's Hospital NHS Foundation Trust Sheffield UK; ^2^ Senior Clinical Lecturer in Neurogenetics & Consultant in Clinical Genetics, Department of Neuroscience The University of Sheffield Sheffield UK

**Keywords:** attitudes, genetics, information, preimplantation genetic diagnosis, prenatal diagnosis, qualitative interviewing, reproductive options

## Abstract

In the United Kingdom (U.K), 2.19 million people are affected by visual loss. Monogenic causes of visual loss include retinal dystrophies, optic neuropathies, and congenital glaucoma. A variety of reproductive options are available to adults with genetic visual loss to permit them to have an unaffected child. Prenatal diagnostic testing (PND) via amniocentesis or chorionic villus sampling (CVS) or Preimplantation Genetic Testing (PGT) is possible, provided the causal genetic variants are known in the family. We report a qualitative interview study of people with genetic causes of visual loss to explore their attitudes toward reproductive options. Participants reported a range of challenges associated with living with genetic conditions associated with visual loss. These had the potential to shape attitudes to reproductive options. Participants expressed enthusiasm for genetic testing, as it enabled them to understand if relatives might be affected by the visual loss. Decisions around reproductive options were recognized as challenging and highly personal. Positive opinions of PGT were reported, as it permitted conception of a child without the genetic cause of visual loss while avoiding the need for the termination of pregnancy. The provision of accessible information resources on genetics and reproductive options was reported to be important.

## Graphical abstract

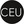

**What is known about this topic**

Little is known about the attitudes of people with genetic visual loss toward reproductive options such as preimplantation genetic testing.

**What does this article add to the topic**

People with genetic visual loss value access to genetic testing and accessible information on reproductive options.

1

In the United Kingdom (U.K), 2.19 million people are affected by visual loss (Galvin et al., 2020). Monogenic causes of visual loss include retinal dystrophies, optic neuropathies, and congenital glaucoma. Globally, it is estimated that 1: 1380 people are affected by an autosomal‐recessive retinal dystrophy with 1: 8000 affected by autosomal‐recessive retinitis pigmentosa (R.P) (Hanany et al., 2020; Jaffal et al., 2021). In the U.K, around 1:4000 people are affected by R.P and 30% of these are autosomal dominant (Daiger et al., 2014). Despite their rarity, the impact of genetic visual loss is substantial. In the U.K, the economic cost for inherited retinal dystrophies is £523 million per annum (Galvin et al., 2020).

A variety of reproductive options are available to adults with genetic visual loss to permit them to have an unaffected child. Prenatal diagnostic testing (PND) via amniocentesis or chorionic villus sampling (CVS) – with potential termination of affected pregnancies – is possible, provided the causal genetic variants are known in the family (Sciorio et al., 2021). Genetic causes of visual loss meet the U.K. National Health Service (N.H.S.) criteria for preimplantation genetic testing (PGT), since it is a serious genetic disorder with >10% chance of transmission (Sciorio et al., 2021).

There is a growing literature on the attitudes of people with genetic conditions toward reproductive options. There is evidence that some people with genetic conditions feel a moral obligation to utilize reproductive options to avoid having an affected child (Decruyenaere et al., 2007). Conversely, other studies suggest that some with a genetic condition integrate having the genetic condition into their identity and do not view the condition negatively (Boardman, 2014; Boardman & Hale, 2018). There is also a lack of consensus between patients and clinicians as to what constitutes a severe medical condition (Boardman & Clark, 2021). Little is known about the opinions of people with genetic causes of visual loss toward reproductive options. We report a qualitative interview study of people with genetic causes of visual loss, to explore their attitudes toward reproductive options.

A purposeful sampling strategy was used to recruit consecutive participants >18 years old who had a diagnosis of a genetic eye condition and received clinical genetic counseling (Table S1). Research Ethics approval was granted by Leeds East Research Ethics Committee (16/YH/0026). Written informed consent was obtained.

The attitudes of adults with genetic causes of visual loss toward reproductive options were explored using an inductive qualitative design for semi‐structured interviews (interview schedule in supplemental Data  S1). The Standards for the Reporting of Qualitative Research guidelines were followed. The interview schedule was developed by a single investigator (AM). Topics were selected based upon a survey conducted with five clinicians (questionnaire distributed by email to two clinical geneticists and three genetic counselors) to ascertain what should be discussed in a clinical consultation for genetic eye disease. This identified mode of inheritance, discussion of reproductive options (PND, PGT), and provision of written information as priorities. Interviews were performed by a single investigator (AM) between March 2020 and April 2021, audio recorded (with consent), and transcribed verbatim. The analysis took place between November 2020 and August 2021. Nvivo 12 was used for analysis. Line by line coding of the transcripts was undertaken, by one investigator (SR). A second investigator (AM) repeated and reviewed the coding on the transcripts to achieve consensus on code definition. 'Charting' was then performed to summarize interview data by code for each participant and thematic analysis was undertaken.

Twenty‐five participants were invited and 17 (68%) responded. One‐on‐one interviews were undertaken with 17 participants (eight females [47%], mean age 37 ± 14 years). Thirteen participants had children, but none had used PND or PGT. Interview length ranged from 15 to 41 min (mean 27 min). The most common cause of visual loss was RP (11/17, 65%). Four main themes were identified.

1.1

*Theme 1. Challenges of living with visual loss*

Participants had experienced a spectrum of physical, psychosocial, and emotional challenges associated with visual loss. These had the potential to influence attitudes to reproductive options.

P3: “I remember going to a parents' evening at the school and finding, you know, when it wasn't lit and there were steps, I had to really take care going down” (male, 60).

Additionally, participants reported being unable to partake in a number of social activities due to restrictions imposed by their visual loss, especially at night.

P3: “I couldn't go running in an evening or join people that are going on an evening run or an evening walk” (male, 60).

Gaining an education was perceived as difficult by the majority of participants. Participants reported a lack of support and understanding from teachers.

P14: “I was very attentive, so it didn't really affect me in the way like I had bad grades, just it was difficult for me to see the board, so I had to concentrate a lot more than most people. Later on, I started having headaches because l was squinting all day long at school and university, which was tiring” (female, 30).

Participants were then asked how they felt, or would feel, about having a child with visual loss. Some participants were clear that they would not want their child to experience what they had.

P2: “I wasn't sure if it was fair for me to have children, because I know what I've been through, and I know what it's like […] I didn't think it was fair to knowingly put that on a child” (female, 30).

1.2

*Theme 2. Understanding of inheritance of familial eye condition*

Participants demonstrated an understanding of the fundamentals of genetics and the inheritance pattern of their eye condition.

P7: “From my understanding, if I became pregnant, if I was having a girl there would be a 50% chance, she'd be completely non‐affected and a 50% chance that she'd be a carrier like me and then if it was a boy, he'd either be completely affected with RP or completely non‐affected like my brother” (female, 25).

Almost all participants expressed enthusiasm and confirmed their support for clinical genetic testing. The potential benefits of testing were highly regarded and acted as a source of motivation to pursue testing. The ability to gain information was seen as paramount.

P10: “If you know what's going to happen to you, then you're more likely to accept it and grow with it. […] It helped us to grow from that and I do believe that if you know what's going to face you, then you've got a better chance to cope with things” (female, 35).

1.3

*Theme 3. Ambivalent reproductive choices*

Many participants inextricably associated PND with the termination of an affected pregnancy. As a result, their negative attitudes toward PND were based on their opinions of termination.

P16: “Baby has the right to live, you know […] I've been successful in my life, and I can secure a good life for them, and I think they can succeed, and they have the right to live you know. That's something that I really fight for” (male, 35).

Some participants reflected that if PND had been used in their family, then they or their family members may not have been born and lived the lives that they have. This generated feelings of confusion and melancholy, as participants felt that PND implies that their life with a genetic condition is undesirable.

P4: “If they answered yes to terminate a pregnancy with RP, then somebody like me wouldn't have ever had a life and lived a life like I've had, I think I've had quite a full life. But then you've got the other side of the coin where you say, well, let's terminate the pregnancy and then the baby doesn't have all the struggles of suffering with RP, growing up with it and growing old with it and the limitations it places on someone's life” (male, 50).

It was common for PGT to be viewed more positively and to be considered more ethical and moral than PND because it does not involve TOP.

P7: “This [PGT] is something I would definitely be considering and willing to try, because as opposed to amniocentesis, it is just the basic cells… so, I know that's a lot more ethical in my opinion” (female, 25).

PGT was also considered psychologically better than PND, since the procedure is carried out prior to pregnancy and eliminates the worry of having an affected pregnancy and child.

P14: “The fact that psychologically it [PGT] is better, generally it is all before you are pregnant” (female, 30).

The opportunity for couples to conceive an unaffected, biologically related child was paramount. Avoiding the chance of passing on the condition to future generations, and preventing future children experiencing the burdens of living with a visual loss, was seen as desirable.

P10: “I think it is acceptable, I know the world's changing at the end of the day and anything… I wouldn't like to say it's making the perfect child because there is no perfect anywhere, but if it stops pain, if it stops the upset, then I'm all for it, yes” (female, 35).

Various logistical and safety concerns were raised with PGT.

P6: “It just feels daunting and risky […] the actual getting of the eggs… and reimplanting it back into me… all the injections because I really don't like needles… and just the fact you could go through all of it, and it not take […] I don't know how many eggs you'd implant but obviously the more eggs you implanted the more chance it would take, but then you could end up with a multiple pregnancy and we'd have to move house” (female, 22).

Theme 4. The need for accessible health information.

Making reproductive decisions requires access to reliable information. Current NHS information on leaflets is not designed for people with visual loss. However, some participants could access the leaflets through the use of adaptive technology.

P4: “I use monomouse, so it magnifies it on a TV screen” (male, 50).

To improve accessibility, participants suggested making text large, bold, and high contrast, as well as using layman's terminology to explain concepts and processes. Suggestions of audio‐ and video‐based information were also made.

P4: “Information wise, it would probably better in high contrast and large print. For me, most things need to be in large print,” (male, 50).

The majority of participants used the Internet on their smartphones to access health information. Smartphone technology allowed them to customize the presentation of information to their preference, such as enlarging the text or inverting the colors.

P2: “My phone talks and my laptop talks… anything that I can access via email, word documents or the Internet, the only thing that isn't accessible are things that are scanned in or printed material like leaflets” (female, 30).

Participants strongly supported that all people with a genetic cause of visual loss should be given comprehensive information on available reproductive options.

P4: “I think people should be made aware of what options are available to them and what the possibilities could be. […] I think put forward all the options that are available and realistically achievable” (male, 50).

The preferred approach for obtaining information was face‐to‐face clinics. Combining clinics with accessible online information was described as optimal.

P1: “I think talking to somebody and having the resources to do it online, to read up on it online. I think both options are really good because it is good to talk to someone about this” (female, 35).

We report the attitudes of people with genetic visual loss toward reproductive options. We confirm the negative impact of genetic causes of visual loss on day‐to‐day life (Anil & Garip, 2018). Despite the negative impact of visual loss, most participants described ways of adapting and leading fulfilling lives. This is reflected in the ambivalent opinions on reproductive options expressed by participants. The wish to have a child unaffected by an eye condition was described, but also recognition that a person with visual loss could have a good quality of life; and that this would be lost if reproductive options had been used. Our participants shared the 'expressivist objection' that PND and termination devalues those with a disability (Felicity Kate Boardman, 2014). This has been reported in other genetic conditions, such as spinal muscular atrophy (SMA), and can influence reproductive decision‐making (Felicity Kate Boardman, 2014). Despite this, participants expressed a desire for information on reproductive options to enable informed decision‐making. It must be recognized that none of our participants had utilized PND or PGT. This is a major limitation since our findings reflect their attitudes toward the hypothetical use of such reproductive options and are not informed by personal experience. Individuals who had utilized PND or PGT may report different attitudes based on positive or negative experiences.

In a U.K. qualitative interview study, adults with inherited retinal dystrophies were reported to have negative opinions of PND (Ahmed et al., 2015). A survey of 200 people with inherited retinal diseases found that 52% would support genetic testing to permit PGT and Forty‐five percent supported PND (Willis et al., 2013). These findings reflect the results of our interview study. Many of our participants objected to terminating pregnancies affected by a genetic cause of visual loss. In general, participants were supportive of PGT and PND being available to people with genetic visual loss but felt they might be unlikely to use these techniques themselves. The attitudes toward reproductive options in our study show similarities to those described in other genetic conditions (Shkedi‐Rafid et al., 2021). For example, some with Huntington's disease report feeling morally obliged to prevent the inheritance of the condition by their children, with PND and PGT being viewed as acceptable (Decruyenaere et al., 2007).

We propose some practical steps to improve access of people with genetic visual loss to reproductive options advice. Clinicians must not assume people with a genetic visual loss will not want reproductive option information. Relevant reproductive options should be discussed with them. Clinicians should be aware that people with genetic visual loss may find discussions of reproductive options challenging (due to, for example, a desire to have an unaffected child while recognizing the value of people with visual loss), and provide sensitive genetic counseling. Our findings suggest that an in‐person discussion in the clinic, supported with information resources, might be the most appropriate way of providing information on reproductive options. Current information resources on reproductive options are not accessible to those with visual loss. Specially designed information resources on reproductive options for people with genetic visual loss should be developed. Discussion of reproductive options in the clinic with the provision of accessible information resources was deemed appropriate. Future research could explore how best to design resources to support informed reproductive decision‐making in people with genetic causes of visual loss.

## AUTHOR CONTRIBUTIONS

**Sophie Redgrave:** Investigation; writing – original draft. **Alisdair Mcneill:** Conceptualization; writing – original draft.

## COMPLIANCE WITH ETHICAL STANDARDS

### Conflict of interest

We have no financial or other conflicts of interest to disclose.

### Human studies and informed consent

Research Ethics approval was granted by the Leeds East Research Ethics Committee (16/YH/0026). Written informed consent was obtained. The Declaration of Helsinki was followed.

### Animal studies

No animal research undertaken.

### Data sharing and data accessibility

Due to the potential to identify participants, we will not make data available.

## Supporting information


Table S1

Data S1

## References

[jgc41601-bib-0001] Ahmed, K. , Ahmed, M. , Potrata, B. , Willis, T. A. , Grant, H. L. , Allsop, M. J. , Hewison, J. , Downey, L. , Gale, R. , & Mckibbin, M. (2015). Patient attitudes towards prenatal diagnostic testing for inherited retinal disease. Prenatal Diagnosis, 35(9), 913–918. 10.1002/pd.4644 26126503

[jgc41601-bib-0002] Anil, K. , & Garip, G. (2018). Coping strategies, vision‐related quality of life, and emotional health in managing retinitis pigmentosa: A survey study. BMC Ophthalmology, 18(1), 21. 10.1186/s12886-018-0689-2 29378559 PMC5789612

[jgc41601-bib-0003] Boardman, F. K. , & Clark, C. C. (2021). What is a 'serious' genetic condition? The perceptions of people living with genetic conditions. European Journal of Human Genetics, 30(2), 160–169. 10.1038/s41431-021-00962-2 34565797 PMC8821585

[jgc41601-bib-0004] Boardman, F. K. , & Hale, R. (2018). How do genetically disabled adults view selective reproduction? Impairment, identity, and genetic screening. Molecular Genetics and Genomic Medicine, 6(6), 941–956. 10.1002/mgg3.463 30196552 PMC6305648

[jgc41601-bib-0005] Boardman, F. K. (2014). The expressivist objection to prenatal testing: The experiences of families living with genetic disease. Social Science and Medicine, 107, 18–25. 10.1016/j.socscimed.2014.02.025 24602967

[jgc41601-bib-0006] Daiger, S. P. , Bowne, S. J. , & Sullivan, L. S. (2014). Genes and Mutations Causing Autosomal Dominant Retinitis Pigmentosa. Cold Spring Harbor Perspectives in Medicine, 5(10), 1–14. 10.1101/cshperspect.a017129 PMC4588133 25304133

[jgc41601-bib-0007] Decruyenaere, M. , Evers‐Kiebooms, G. , Boogaerts, A. , Philippe, K. , Demyttenaere, K. , Dom, R. , Vandenberghe, W. , & Fryns, J. P. (2007). The complexity of reproductive decision‐making in asymptomatic carriers of the Huntington mutation. European Journal of Human Genetics, 15(4), 453–462. 10.1038/sj.ejhg.5201774 17245406

[jgc41601-bib-0008] Galvin, O. , Chi, G. , Brady, L. , Hippert, C. , Rubido, M. D. V. , Daly, A. , & Michaelides, M. (2020). The impact of inherited retinal diseases in the Republic of Ireland (ROI) and the United Kingdom (UK) from a cost‐of‐illness perspective. In. Clinical Ophthalmology, 14, 707–719. 10.2147/OPTH.S241928 32184557 PMC7062501

[jgc41601-bib-0009] Hanany, M. , Rivolta, C. , & Sharon, D. (2020). Worldwide carrier frequency and genetic prevalence of autosomal recessive inherited retinal diseases. Proceedings of the National Academy of Sciences of the United States of America, 117(5), 2710–2716. 10.1073/pnas.1913179117 31964843 PMC7007541

[jgc41601-bib-0010] Jaffal, L. , Joumaa, H. , Mrad, Z. , Zeitz, C. , Audo, I. , & El Shamieh, S. (2021). The genetics of rod‐cone dystrophy in Arab countries: a systematic review. European Journal of Human Genetics, 29(6), 897–910. 10.1038/s41431-020-00754-0 33188265 PMC8187393

[jgc41601-bib-0012] Sciorio, R. , Aiello, R. , & Irollo, A. M. (2021). Review: Preimplantation genetic diagnosis (PGD) as a reproductive option in patients with neurodegenerative disorders. Reproductive Biology, 21(1), 100468. 10.1016/j.repbio.2020.100468 33321391

[jgc41601-bib-0013] Shkedi‐Rafid, S. , Horton, R. , & Lucassen, A. (2021). What is the meaning of a 'genomic result' in the context of pregnancy? European Journal of Human Genetics, 29(2), 225–230. 10.1038/s41431-020-00722-8 32929236 PMC7868356

[jgc41601-bib-0014] Willis, T. A. , Potrata, B. , Ahmed, M. , Hewison, J. , Gale, R. , Downey, L. , & McKibbin, M. (2013). Understanding of and attitudes to genetic testing for inherited retinal disease: A patient perspective. British Journal of Ophthalmology, 97(9), 1148–1154. 10.1136/bjophthalmol-2013-303434 23813418 PMC3756432

