# Supplementary material for: A qualitative interview study of the attitudes toward reproductive options of people with genetic visual loss
Source: J Genet Couns. 2022 Jul 4;31(5):1231–4. doi: 10.1002/jgc4.1601 (PMC9796805; doi:10.1002/jgc4.1601)
Supplement: Supplementary file 1 — Table S1 [file JGC4-31-1231-s001.docx]

**Supplementary Table 1. Demographic and clinical characteristics of participants.**

|  | Demographics  Age, sex | Diagnosis | Relative affected by same condition |
| --- | --- | --- | --- |
| Participant 1 | 35, F | Congenital stationary night blindness | Yes |
| Participant 2 | 30, F | FEVR | No |
| Participant 3 | 60, M | Retinitis pigmentosa | Yes |
| Participant 4 | 50, M | Retinitis pigmentosa | Yes |
| Participant 5 | 55, M | Retinitis pigmentosa | Yes |
| Participant 6 | 22, F | Autosomal recessive albinism | No |
| Participant 7 | 25, F | Retinitis pigmentosa | Yes |
| Participant 8 | 30, M | Leber congenital amaurosis | No |
| Participant 9 | 70, M | Retinitis pigmentosa | Yes |
| Participant 10 | 35, F | Congenital stationary night blindness | Yes |
| Participant 11 | 35, M | Optic atrophy | Yes |
| Participant 12 | 30, M | Retinitis pigmentosa | No |
| Participant 13 | 50, M | Retinitis pigmentosa | Yes |
| Participant 14 | 30, F | Wagner syndrome | Yes |
| Participant 15 | 50, F | macular dystrophy | No |
| Participant 16 | 35, M | Retinitis pigmentosa | No |
| Participant 17 | 20, F | Peters anomaly | No |
