# Supplementary material for: A qualitative interview study of the attitudes toward reproductive options of people with genetic visual loss
Source: J Genet Couns. 2022 Jul 4;31(5):1231–4. doi: 10.1002/jgc4.1601 (PMC9796805; doi:10.1002/jgc4.1601)
Supplement: Supplementary file 2 — Data S1 [file JGC4-31-1231-s002.docx]

Supporting information

Methods – Interview Schedule

Indicative qualitative interview schedule

Opening

(Introductions) - [Greet participant/volunteer, check environmental factors/comfort, check what they prefer to be called, introduce self and role]

I would like to ask you some questions about your eye condition, your understanding of genetic testing in eye conditions, and your views about reproductive medicine options in eye conditions. First, I will explain more about the study and this interview. Please let me know if you have questions at any point.

(Purpose) - I hope to use the information, we gather as part of a research study exploring people's views and feelings about reproductive options in genetic eye conditions, and we hope the information we collect will feed into recommendations for genetic eye conditions. Therefore, it would be helpful if you could share as much as feels comfortable for you.

(Time) - My estimate is that this interview could take around 30–60 minutes. However, it could be more or less depending on your communication style and how much you have to say in response to each question. We can take as many breaks as you need to.

Confirm background details of interviewee – age, employment status, who in the family is affected by the eye condition.

1. Main interview topics
2. I would like to start by asking you to tell me about your understanding of your genetic eye condition?

- How did you find out you had the condition?
- Prompts to ask about symptoms, natural history, treatments, and impact on the family.

1. Can you tell me about your understanding of the genetics of your eye condition?

- Understanding of genes and chromosomes

1. Based upon your understanding of the eye condition is anyone else in the family at risk of having the condition?

- Can it pass from mum/dad to child?
- Do you know what the chance is?
- What are your views on having a family?

1. How was the information on inheritance and genetics shared in your family?

- Prompts: how did they learn about genetic inheritance of it? Have they told anyone?

1. Have you heard of any pregnancy options which can ensure that a baby is born without a specific genetic condition?

[**if they have not**: explain CVS and amniocentesis]

- Prompts: have they heard of prenatal diagnosis (CVS, amniocentesis)?
- Have they heard of non-invasive prenatal testing (NIPT)? What do they think of it.

1. Have you heard of a treatment called preimplantation genetic diagnosis?

[**if they have not explain**: a form of IVF, where sperm and egg are combined in a laboratory to make an embryo, this is then tested for the 22q11 deletion and only embryos without the deletion are placed in the womb]

- Prompts: why do you find, for example, PGD acceptable/unacceptable?
- Do you think PGD should be offered/discussed with people with 22q11DS?
- Do you think PGD should be offered on the NHS for people with certain genetic conditions?

1. What do you think of the currently available information leaflets on PND/PGD?

- Prompts: has PGD, etc, been discussed with them in the past? By whom? Have they seen any information leaflets and what did they think?

1. How would you prefer to get information on PND/PGD/NIPT?

- What type format of leaflet would you like? Heard of infographics?
- What is your view of Internet-based/smartphone-based resources for information?
- What problems accessing/understanding information do they have?

1. What information do you think people with genetic eye conditions should be told about their chances of passing it on to their children?
2. What are your views on people with genetic eye conditions being made aware of pregnancy options such as PND/PGD/NIPT?
3. Close

I have now asked all of the planned questions. Is there anything else you think would be helpful for me to know about your experiences or your views? Do you have any questions for me?

Thank you very much for taking part in our study. If you have any questions after leaving here today, please feel free to contact the study team. Our contact details are on the information leaflet that I provided.
